# Pathways and genes differentially expressed in the motor cortex of patients with sporadic amyotrophic lateral sclerosis

**DOI:** 10.1186/1471-2164-8-26

**Published:** 2007-01-23

**Authors:** Carsten W Lederer, Antonietta Torrisi, Maria Pantelidou, Niovi Santama, Sebastiano Cavallaro

**Affiliations:** 1 Department of Biological Sciences, University of Cyprus and Cyprus Institute of Neurology and Genetics, 1678 Nicosia, Cyprus; 2 Functional Genomics Center, Institute of Neurological Sciences, Italian National Research Council, 95123 Catania, Italy

## Abstract

**Background:**

Amyotrophic lateral sclerosis (ALS) is a fatal disorder caused by the progressive degeneration of motoneurons in brain and spinal cord. Despite identification of disease-linked mutations, the diversity of processes involved and the ambiguity of their relative importance in ALS pathogenesis still represent a major impediment to disease models as a basis for effective therapies. Moreover, the human motor cortex, although critical to ALS pathology and physiologically altered in most forms of the disease, has not been screened systematically for therapeutic targets.

**Results:**

By whole-genome expression profiling and stringent significance tests we identify genes and gene groups de-regulated in the motor cortex of patients with sporadic ALS, and interpret the role of individual candidate genes in a framework of differentially expressed pathways. Our findings emphasize the importance of defense responses and cytoskeletal, mitochondrial and proteasomal dysfunction, reflect reduced neuronal maintenance and vesicle trafficking, and implicate impaired ion homeostasis and glycolysis in ALS pathogenesis. Additionally, we compared our dataset with publicly available data for the SALS spinal cord, and show a high correlation of changes linked to the diseased state in the SALS motor cortex. In an analogous comparison with data for the Alzheimer's disease hippocampus we demonstrate a low correlation of global changes and a moderate correlation for changes specifically linked to the SALS diseased state.

**Conclusion:**

Gene and sample numbers investigated allow pathway- and gene-based analyses by established error-correction methods, drawing a molecular portrait of the ALS motor cortex that faithfully represents many known disease features and uncovers several novel aspects of ALS pathology. Contrary to expectations for a tissue under oxidative stress, nuclear-encoded mitochondrial genes are uniformly down-regulated. Moreover, the down-regulation of mitochondrial and glycolytic genes implies a combined reduction of mitochondrial and cytoplasmic energy supply, with a possible role in the death of ALS motoneurons. Identifying candidate genes exclusively expressed in non-neuronal cells, we also highlight the importance of these cells in disease development in the motor cortex. Notably, some pathways and candidate genes identified by this study are direct or indirect targets of medication already applied to unrelated illnesses and point the way towards the rapid development of effective symptomatic ALS therapies.

## Background

Amyotrophic lateral sclerosis (ALS) is a fatal neuromuscular disorder affecting 1–2 in 100,000 persons. It is caused by the degeneration of motoneurons in brain and spinal cord, leading to muscle atrophy, progressive paralysis, and death, commonly by respiratory failure. Most cases of ALS are sporadic (SALS) and about 10% familial (FALS), with mutant forms of copper-zinc superoxide dismutase (SOD1) causing 20% of FALS cases. Findings in ALS patients and model systems have implicated numerous genes in ALS pathogenesis, and have identified diverse processes, such as oxidative stress, excitotoxicity, mitochondrial dysfunction, protein aggregation, cytoskeletal abnormalities, impaired axonal transport, inflammation, and apoptosis, as contributing factors [1]. As a mainly sporadic disease affecting multiple cellular processes, ALS therefore suggests itself for comprehensive expression profiling and gene- and pathway-based analyses. The few existing genomics studies of ALS [2-4], however, have had limited genome coverage and have been restricted to gene-based analyses.

Adding a further layer of complexity, ALS is a highly heterogenous disease, with clinical indicators helping to define ALS subtypes. One such indicator is a differential depletion of motoneurons in motor cortex and spinal cord [5-7], giving significance to the characterization of both tissues. The motor cortex contains upper motoneurons (UMNs), which extend axons traversing the corticospinal tract to signal to the spinal cord, where lower motoneurons (LMNs) relay their signal. The corticospinal tract volume is reduced [8] and UMNs are depleted [9,10] in ALS patients, and UMNs are required for LMN function and muscle control [11]. Most importantly, patients with sporadic, non-SOD1-associated forms of ALS show alterations in the motor cortex, such as increased excitability and reduced inhibitory activity, which are not readily detectable in SOD1-linked FALS patients, thus stressing the particular importance of investigating the motor cortex in SALS subjects [12-14]. Possibly owing to earlier clinical manifestations of defects in LMNs and their easier accessibility to experimenters, however, most investigations of ALS, including previous genomics studies [2-4], focus on the spinal cord and LMNs, leaving ALS-related responses and defects of cellular maintenance in the motor cortex under-investigated.

Here we address the importance of UMN abnormalities in SALS pathology and exploit the power of pathway-based significance tests by whole-genome expression profiling of the motor cortex of SALS patients. We identify differentially expressed genes and pathways, interpret the role of candidate genes in ALS pathology using these pathways as a functional outline, and evaluate the implications of our findings for ALS research and the development of ALS therapies.

## Results

### Expression profiling of the motor cortex of SALS patients

**Figure 1:**
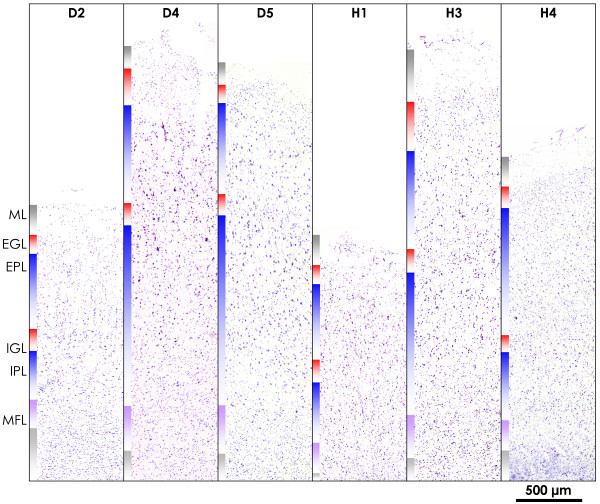
**Architecture of the motor cortex in SALS and control samples**. Nissl staining of representative SALS samples (D2, D4, D5) and controls (H1, H3, H4) shows the intact architecture of the motor cortex in samples chosen for expression analysis. All samples are shown at identical magnification. Cell layers are color-coded as indicated for sample D2. ML – molecular layer; EGL – external granular layer; EPL – external pyramidal layer; IGL – internal granular layer; IPL – internal pyramidal layer; MFL – multiform layer; WM – white matter. The scale bar indicates 500 μm.

We investigated the motor cortex of eleven SALS and nine control subjects (see Table 1) with whole-genome oligonucleotide microarrays and following microscopic assessment of tissue architecture (see Figure 1 for Nissl staining of six representative samples). Out of over 41,000 genes and expressed sequence tags tested, 19,431 genes passed our quality control criteria (see the *Methods *section), constituting our complete data set for subsequent analyses. Median and average fold-changes of our complete data set were 1.01 (Diseased vs. Control), establishing that no systematic bias for up- or down-regulation was introduced into subsequent statistical analyses.

**Table 1:** Characteristics of subjects

**Sample ID**	**Sample Type**	**Order Code**	**Chief Disease**	**Cause of Death**	**Age [years/days]**	**Gender**	**Race**	**PMI [h]**
Control 1	tissue	BTB3428 ^A^	drowning	drowning	56/275	Female	Caucasian	17
Control 2	tissue	BTB3576^A^	hypertension	ruptured abdominal aortic aneurism	59/260	Male	Caucasian	15
Control 3	tissue	BTB3577^A^	n/a	n/a	59/118	Male	Caucasian	21
Control 4	tissue	BTB3688^A^	ascending colon adenocarcinoma	ascending colon adenocarcinoma	60/-	Male	Hispanic White	17
Control 5	tissue	HCtAN^A^	n/a	n/a	72/-	Male	Caucasian	11
Control 6	tissue	HCtAV^A^	n/a	n/a	84/-	Female	Caucasian	2.75
Control 7	tissue	HCtEU^A^	coronary artery disease	n/a	79/-	Female	Caucasian	7.5
Control 8	tissue	HCtFM^A^	n/a	n/a	70/-	Female	Caucasian	8.5
Control 9	RNA	6782^B^	thrombosis and coronary atherosclerosis	heart attack	68/-	Male	Caucasian	n/a
Diseased 1	tissue	BTB3226 A	ALS	respiratory failure with probable early pneumonia	71/18	Male	Caucasian	19
Diseased 2	tissue	BTB3235 A	ALS	respiratory failure	73/106	Male	Caucasian	5.5
Diseased 3	tissue	BTB3461 A	ALS	ALS	77/53	Male	Caucasian	7.5
Diseased 4	tissue	BTB3580^A^	ALS	respiratory failure	58/83	Male	Caucasian	22
Diseased 5	tissue	BTB3583^A^	ALS	respiratory distress	52/54	Female	Hispanic White	20.5
Diseased 6	tissue	BTB3590^A^	ALS	pneumonia	75/-	Male	Caucasian	24
Diseased 7	tissue	BTB3603^A^	ALS	N/A	67/325	Female	Caucasian	17
Diseased 8	tissue	BTB3611^A^	ALS	N/A	69/90	Female	Caucasian	13
Diseased 9	tissue	BTB3689^A^	ALS	N/A	61/141	Female	Caucasian	22
Diseased 10	tissue	BTB3713^A^	ALS	ALS	66/252	Male	Caucasian	13
Diseased 11	RNA	6166^B^	ALS	respiratory failure	70/-	Male	Caucasian	n/a

20 motor-cortex samples of eleven ALS patients and nine controls were analyzed on individual Whole Human Genome Arrays for genome-wide expression profiling. Age, gender, race, and post-mortem interval (mean controls 12.47 ± 6.06 h and mean patients 16.35 ± 6.37 h, *P *= 0.21) are shown for all tissue samples. A – Miami Brain and Tissue Bank for Developmental Disorders; B – Ambion, Inc.; n/a – not available

To exploit the comprehensiveness of our data, we combined two complementary, stringent approaches, investigating changes in the SALS motor cortex for functional gene groups and on the level of individual genes.

### Pathway-based analysis

**Figure 2:**
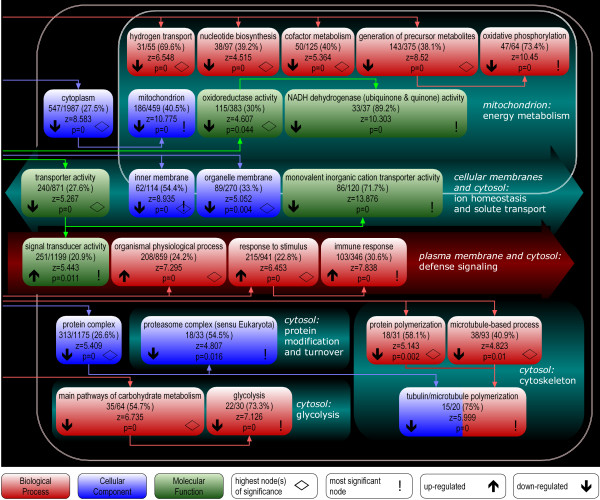
**Gene Ontology nodes differentially expressed in motor cortex of SALS subjects**. Significant Gene Ontology (GO) nodes were combined to groups likely representing major processes in the context of ALS pathology, specifically *energy metabolism*, *ion homeostasis and solute transport*, *defense signaling*, *protein modification and turnover*, *cytoskeleton*, and *glycolysis*, and are arranged indicating the predominant intracellular localization of the gene products they comprise. The basic categories Biological Process, Cellular Component, and Molecular Function are color-coded. Connecting arrows indicate the order of nodes in the GO hierarchy. For each major process only the highest node(s) of significance (◇) and the most significant node (!) are shown per basic category. Up- () and down-regulation (), the absolute number and percentage of genes meeting the criterion, z score, and corrected *P *value are indicated for each node. Redundant nodes are left out within the same category, or combined if in different categories.

We first performed a pathway-based analysis of the complete data set by testing changes of gene expression in the context of 5137 gene groups of the gene ontology (GO) hierarchy, using the GenMAPP 2.0 software. Analyses on the level of functional gene groups lead to a biologically transparent picture of expression, accessible to interpretation. What is more, by combining measurements for functionally related genes this approach increases the sensitivity of detecting significant changes when differential gene expression is diluted in a mixed cell population (leading to comparably low fold changes) and with small to moderate sample numbers (possibly leading to a high variance for inconsistently de-regulated genes). In such instances individual genes will not pass statistical criteria for differential gene expression, while moderate deregulation of multiple genes constituting a functional group might test highly significant. Indeed, the statistical significances we derived from GenMAPP 2.0, using the average fold-change per gene as a basis for statistical tests, is independent of the number of samples and highly dependent on the number of genes represented by our data, while the quality filtering and the comparably large number of samples used in this study avoid the detection of spurious results and promote a correspondence between statistical and biological significance. In other words, 5137 GO categories were probed with 19,431 quality filtered genes, and 20 samples were used to derive representative average fold-changes to do so. GenMAPP detected 5033 GO categories as unchanged, while 104 partially redundant categories exhibited significantly changed expression in ALS subjects (corrected *P *< 0.05). Significant GO categories fell into six major processes of relevance in the context of a comprehensive ALS disease model, five of them down-regulated and one up-regulated (Figure 2; see Table 2 for the full list of significantly changed GO categories).

**Table 2:** Differentially regulated GO categories

**Up-regulated GO categories**							
**GO ID**	**GO Name**	**GO Type**	**Number Changed**	**Number Measured**	**Percent Changed**	**z Score**	**corrected *P***
6955	immune response	BP	106	346	30.64	7.84	0
9607	response to biotic stimulus	BP	130	456	28.51	7.77	0
6952	defense response	BP	113	381	29.66	7.71	0
50874	organismal physiological process	BP	208	859	24.21	7.30	0
50896	response to stimulus	BP	215	941	22.85	6.45	0
43207	response to external biotic stimulus	BP	71	235	30.21	6.24	0
9613	response to pest\, pathogen or parasite	BP	69	228	30.26	6.16	0
9605	response to external stimulus	BP	134	563	23.80	5.51	0.01
4871	signal transducer activity	MF	251	1199	20.93	5.44	0.011
9596	detection of pest\, pathogen or parasite	BP	5	5	100.00	5.19	0.047

2453	probes met the [fold-change]>=1.2 criterion.						
1782	probes meeting the filter linked to an Ensembl ID.						
1323	genes meeting the criterion linked to a GO term.						
The z score is based on an N of 8457 and an R of 1323 distinct genes in the GO.							

**Down-regulated GO categories**							
**GO ID**	**GO Name**	**GO Type**	**Number Changed**	**Number Measured**	**Percent Changed**	**z Score**	**corrected *P***

15077	monovalent inorganic cation transporter activity	MF	86	120	71.67	13.88	0
15078	hydrogen ion transporter activity	MF	80	111	72.07	13.44	0
15399	primary active transporter activity	MF	88	131	67.18	13.23	0
16655	oxidoreductase activity\, acting on NADH or NADPH\, quinone or similar compound as acceptor	MF	37	42	88.10	10.81	0
5739	mitochondrion	CC	186	459	40.52	10.78	0
3954	NADH dehydrogenase activity	MF	34	38	89.47	10.49	0
6119	oxidative phosphorylation	BP	47	64	73.44	10.45	0
50136	NADH dehydrogenase (quinone) activity	MF	33	37	89.19	10.30	0
8137	NADH dehydrogenase (ubiquinone) activity	MF	33	37	89.19	10.30	0
16651	oxidoreductase activity\, acting on NADH or NADPH	MF	43	57	75.44	10.23	0
15081	sodium ion transporter activity	MF	34	40	85.00	10.06	0
19866	inner membrane	CC	62	114	54.39	8.94	0
42775	ATP synthesis coupled electron transport (sensu Eukaryota)	BP	22	24	91.67	8.59	0
5737	cytoplasm	CC	547	1987	27.53	8.58	0
6091	generation of precursor metabolites and energy	BP	143	375	38.13	8.52	0
8324	cation transporter activity	MF	127	324	39.20	8.38	0
42773	ATP synthesis coupled electron transport	BP	22	25	88.00	8.32	0
5740	mitochondrial membrane	CC	59	115	51.30	8.15	0
46873	metal ion transporter activity	MF	42	70	60.00	8.15	0
15075	ion transporter activity	MF	146	397	36.78	8.10	0
5386	carrier activity	MF	109	271	40.22	8.06	0
6120	mitochondrial electron transport\, NADH to ubiquinone	BP	19	21	90.48	7.90	0
5743	mitochondrial inner membrane	CC	48	89	53.93	7.78	0
6096	glycolysis	BP	22	30	73.33	7.13	0
6754	ATP biosynthesis	BP	26	41	63.41	6.77	0
6753	nucleoside phosphate metabolism	BP	26	41	63.41	6.77	0
9206	purine ribonucleoside triphosphate biosynthesis	BP	30	51	58.82	6.74	0
9145	purine nucleoside triphosphate biosynthesis	BP	30	51	58.82	6.74	0
9201	ribonucleoside triphosphate biosynthesis	BP	30	51	58.82	6.74	0
6092	main pathways of carbohydrate metabolism	BP	35	64	54.69	6.74	0
15992	proton transport	BP	31	54	57.41	6.68	0
16818	hydrolase activity\, acting on acid anhydrides\, in phosphorus-containing anhydrides	MF	124	358	34.64	6.65	0
16817	hydrolase activity\, acting on acid anhydrides	MF	124	358	34.64	6.65	0
9142	nucleoside triphosphate biosynthesis	BP	30	52	57.69	6.60	0
15405	P-P-bond-hydrolysis-driven transporter activity	MF	36	68	52.94	6.59	0
9199	ribonucleoside triphosphate metabolism	BP	31	55	56.36	6.55	0
9144	purine nucleoside triphosphate metabolism	BP	31	55	56.36	6.55	0
6818	hydrogen transport	BP	31	55	56.36	6.55	0
9205	purine ribonucleoside triphosphate metabolism	BP	31	55	56.36	6.55	0
46034	ATP metabolism	BP	27	45	60.00	6.52	0
16462	pyrophosphatase activity	MF	122	356	34.27	6.45	0
46961	hydrogen-transporting ATPase activity\, rotational mechanism	MF	23	36	63.89	6.41	0
16469	proton-transporting two-sector ATPase complex	CC	23	36	63.89	6.41	0
9152	purine ribonucleotide biosynthesis	BP	33	62	53.23	6.34	0
9141	nucleoside triphosphate metabolism	BP	31	57	54.39	6.30	0
15985	energy coupled proton transport\, down electrochemical gradient	BP	23	37	62.16	6.24	0
6007	glucose catabolism	BP	23	37	62.16	6.24	0
15986	ATP synthesis coupled proton transport	BP	23	37	62.16	6.24	0
15980	energy derivation by oxidation of organic compounds	BP	46	101	45.54	6.20	0
5489	electron transporter activity	MF	67	168	39.88	6.20	0
45259	proton-transporting ATP synthase complex	CC	10	10	100.00	6.19	0
45255	hydrogen-translocating F-type ATPase complex	CC	10	10	100.00	6.19	0
5753	proton-transporting ATP synthase complex (sensu Eukaryota)	CC	10	10	100.00	6.19	0
46933	hydrogen-transporting ATP synthase activity\, rotational mechanism	MF	22	35	62.86	6.17	0
42625	ATPase activity\, coupled to transmembrane movement of ions	MF	32	61	52.46	6.14	0
19829	cation-transporting ATPase activity	MF	23	38	60.53	6.07	0
17111	nucleoside-triphosphatase activity	MF	114	338	33.73	6.03	0
9150	purine ribonucleotide metabolism	BP	33	65	50.77	6.01	0
6164	purine nucleotide biosynthesis	BP	33	65	50.77	6.01	0
45298	tubulin	BP	15	20	75.00	6.00	0
46785	microtubule polymerization	CC	15	20	75.00	6.00	0
9260	ribonucleotide biosynthesis	BP	33	66	50.00	5.90	0
31109	microtubule polymerization or depolymerization	BP	17	25	68.00	5.84	0
9259	ribonucleotide metabolism	BP	34	70	48.57	5.78	0
46164	alcohol catabolism	BP	23	40	57.50	5.76	0
46365	monosaccharide catabolism	BP	23	40	57.50	5.76	0
19320	hexose catabolism	BP	23	40	57.50	5.76	0
6752	group transfer coenzyme metabolism	BP	27	51	52.94	5.70	0
6163	purine nucleotide metabolism	BP	33	68	48.53	5.69	0
15002	heme-copper terminal oxidase activity	MF	13	17	76.47	5.68	0
16676	oxidoreductase activity\, acting on heme group of donors\, oxygen as acceptor	MF	13	17	76.47	5.68	0
16675	oxidoreductase activity\, acting on heme group of donors	MF	13	17	76.47	5.68	0
4129	cytochrome-c oxidase activity	MF	13	17	76.47	5.68	0
6732	coenzyme metabolism	BP	45	107	42.06	5.49	0
42626	ATPase activity\, coupled to transmembrane movement of substances	MF	36	79	45.57	5.48	0
16820	hydrolase activity\, acting on acid anhydrides\, catalyzing transmembrane movement of substances	MF	36	79	45.57	5.48	0
43234	protein complex	CC	313	1175	26.64	5.41	0
51186	cofactor metabolism	BP	50	125	40.00	5.36	0
6006	glucose metabolism	BP	27	54	50.00	5.33	0
5215	transporter activity	MF	240	871	27.55	5.27	0
45333	cellular respiration	BP	17	28	60.71	5.23	0
51258	protein polymerization	BP	18	31	58.06	5.14	0.002
226	microtubule cytoskeleton organization and biogenesis	BP	25	50	50.00	5.13	0.004
31090	organelle membrane	CC	89	270	32.96	5.05	0.004
7017	microtubule-based process	BP	38	93	40.86	4.82	0.01
502	proteasome complex (sensu Eukaryota)	CC	18	33	54.55	4.81	0.016
16052	carbohydrate catabolism	BP	24	50	48.00	4.78	0.025
44275	cellular carbohydrate catabolism	BP	24	50	48.00	4.78	0.025
9117	nucleotide metabolism	BP	48	128	37.50	4.73	0.026
6118	electron transport	BP	73	220	33.18	4.63	0.043
3924	GTPase activity	MF	44	116	37.93	4.61	0.043
16491	oxidoreductase activity	MF	115	383	30.03	4.61	0.044
5829	cytosol	CC	69	207	33.33	4.54	0.045
9165	nucleotide biosynthesis	BP	38	97	39.18	4.52	0.045

3059	probes met the [fold-change]<=(1/1.2) criterion.						
2522	probes meeting the filter linked to an Ensembl ID.						
1751	genes meeting the criterion linked to a GO term.						
The z score is based on an N of 8457 and an R of 1751 distinct genes in the GO.							

Shown are all GO nodes differentially regulated between SALS motor-cortex-patient samples and controls for the comprehensive dataset. Of 19,431 probes in the dataset, 8,457 were linked to a GO term. Nodes are sorted by up- or down-regulation (top and bottom sub-tables, respectively), then sorted by corrected *P *value (lowest first) and z value (highest first). CC – cellular component; MF – molecular function; BP – biological process

### Identification of candidate genes

**Figure 3:**
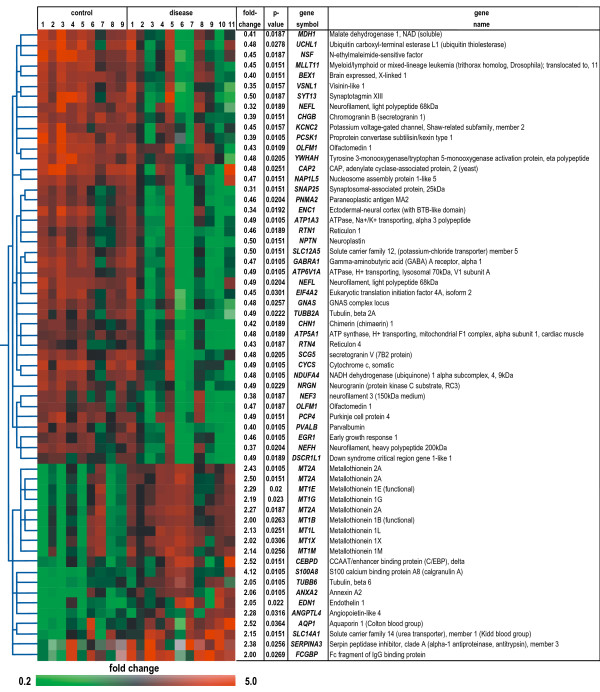
**Genes differentially expressed in motor cortex of SALS subjects**. 57 of 19,431 quality-filtered genes (0.3%), represented by 61 probes, are differentially expressed (corrected *P *< 0.05), with each row in the matrix representing a single probe and each column a subject. Normalized expression levels are represented by the color of the corresponding cell, relative to the median abundance of each gene for each subject (see scale). Genes are named using their UniGene symbol and arranged in a hierarchical cluster (standard correlation) based on their expression patterns, combined with a dendrogram whose branch lengths reflect the relatedness of expression patterns. For each gene the fold-change (diseased vs. control) and corrected *P *value are given.

We then analyzed the complete data set for individual genes showing differential gene expression, finding that 57 of the 19,431 quality-filtered genes (0.3%) fulfilled our stringent criteria (including Benjamini and Hochberg multiple testing correction, for details see the *Methods *section), with 40 genes showing down-regulation and 17 up-regulation in SALS subjects (Figure 3). In contrast to results for large gene groups, differential expression of individual mRNAs is readily verifiable by alternative techniques, such as quantitative RT-PCR (qRT-PCR) and *in-situ *hybridization.

### Confirmation by quantitative RT-PCR

**Figure 4:**
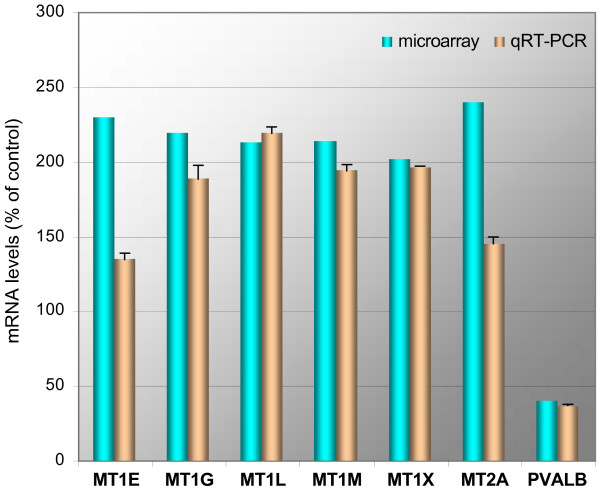
**Comparison of microarray and qRT-PCR results for selected candidate genes**. The bar graph shows mean results of expression levels in SALS vs. control subject. Fold-changes are shown diseased vs. control, and differed by less than 1.2-fold between qRT-PCR and microarray, except for MT1E and MT2A. Error bars for qRT-PCR data indicate the range for duplicate experiments. The *P *values for qRT-PCR and microarray (most significant probe) data were 0.342 and 0.020 (MT1E), 0.063 and 0.023 (MT1G), 0.037 and 0.025 (MT1L), 0.119 and 0.026 (MT1M), 0.074 and 0.031 (MT1X), 0.063 and 0.011 (MT2A), and 0.037 and 0.011 (PVALB).

The 60-mer oligonucleotide format of microarrays used in this study may provide tolerance for sequence mismatches. To investigate probe-target specificity, the sequences of oligonucleotide probes corresponding to differentially expressed genes were compared to GenBank sequences by BLASTN queries. The only probes showing up to ten mismatches with more than one gene were seven metallothioneins (*MT1B*, *MT1E*, *MT1G*, *MT1L*, *MT1M*, *MT1X*, *MT2A*). To discriminate among these homologous genes and analyze their differential expression we used qRT-PCR. PVALB, down-regulated in the ALS motor cortex, was also included in our qRT-PCR analysis as a positive control for the correspondence of microarray and qRT-PCR data. In contrast to the metallothionein probes the PVALB probe, representative of probes for the remaining candidates, detects transcripts for a single gene, only. PVALB and six of the metallothioneins were specifically quantifiable by qRT-PCR (Figure 4). Microarray and qRT-PCR results were concordant (correlation coefficient r = 0.91, comparing individual test and control averages for the seven genes tested), confirming up-regulation of six metallothioneins and down-regulation of PVALB in the SALS motor cortex. *MT1B*, whose microarray probe shares 87% sequence identity with *MT1L*, could not be detected specifically by three independent primer pairs, and was excluded from subsequent analyses.

### Confirmation by *in-situ *hybrization

**Figure 5:**
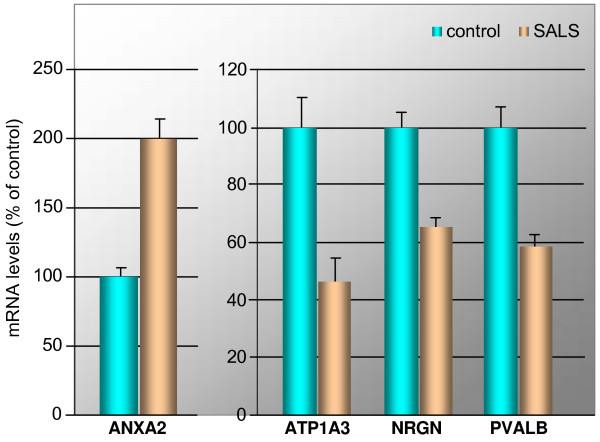
**Changes of ANXA2, ATP1A3, NRGN, and PVALB mRNAs in motor cortex of SALS subjects**. *In-situ *hybridization analysis of ANXA2, ATP1A3, NRGN, and PVALB mRNAs was performed in duplicate tissue sections of motor cortex from five control and five SALS subjects. Optical density values were normalized against those obtained in control subjects and subjected to two-tailed Student's t-test (with the *P *values 3.44e-6 (ANXA2), 7.91e-3 (ATP1A3), 2.29e-5 (NRGN) and 2.88e-6 (PVALB), respectively, diseased vs. control). The bar graph shows mean results and the standard deviation of the mean of labeling in control and SALS subjects.

To verify the microarray results in a direct fashion (i.e. without intervening reverse-transcription and amplification steps), we then performed double-blind *in-situ *hybridization for four candidate genes in human motor cortex sections from five control and five SALS subjects. Median fluorescence levels of entire tissue sections for diseased and control subjects were measured for four candidate genes (*ANXA2*, *ATP1A3*, *NRGN*, and *PVALB*, see Figure 5), showing a strong correlation with the microarray data (correlation coefficient r = 0.97, comparing individual test and control averages for the four genes tested).

### Confirmation at the protein level

**Figure 6:**
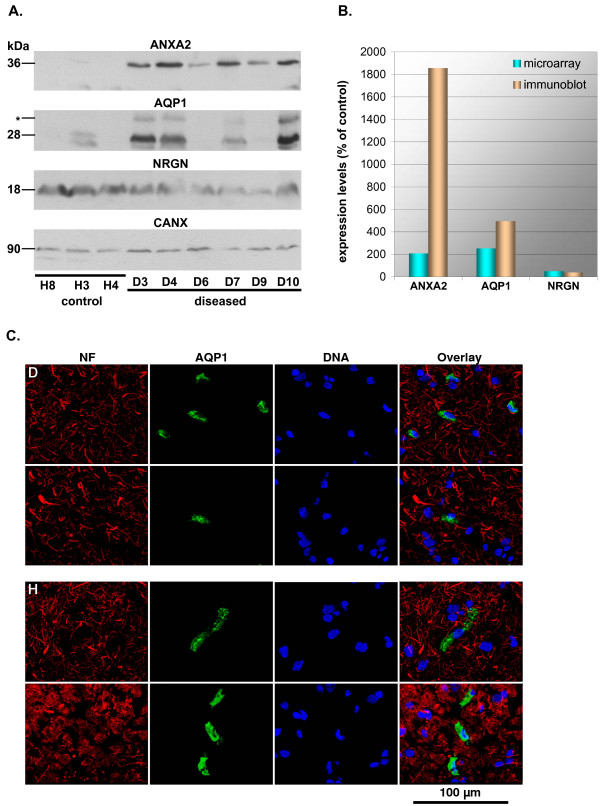
**Immunodetection of selected candidate genes**. (A) Immunoblots for ANXA2, AQP1, NRGN, and CANX was performed for three control and six diseased subjects. The size of quantified bands is indicated in kDa. The glycosylated form of AQP1 (*), undetectable in the healthy samples analyzed, was not included in the analysis. CANX served as a reference gene for sample normalization. (B) Bar graph comparing fold-changes of mean expression levels diseased vs. control as detected by immunoblot and microarray. Normalized intensity values are shown diseased vs. control and were analyzed by a two-tailed Student's t-test. *P *values for immunoblot and microarray (most significant probe) data were 0.025 and 0.011 (ANXA2), 0.055 and 0.036 (AQP1), and 0.047 and 0.023 (NRGN), respectively). (C) Immunofluorescence detection of AQP1 expression in non-neuronal cells in the diseased (D) and the healthy (H) motor cortex. NF – neurofilaments (red); AQP1 – aquaporin 1 (green); DNA – Hoechst33342 counterstain (blue). The scale bar indicates 100 μm.

We then proceeded to validate our findings for mRNAs at the level of proteins, which for the overwhelming majority of RNAs investigated in this study represent the biologically active equivalent. While individual genes may show discrepancies between mRNA and protein levels, owing to translational and post-translational control, an on average fair correlation between protein and mRNA levels can be expected in mammalian systems [15]. Protein extracts of three healthy and six diseased samples, equalized for total protein content, were analyzed by immunoblotting (see Figure 6A and 6B). Quantification of protein levels revealed an up-regulation for ANXA2 (18.56-fold) and AQP1 (4.94-fold), and a down-regulation of NRGN (0.38-fold) in diseased samples. These findings were in line with and exceeded the fold-changes of 2.06, 2.52, and 0.49, respectively, detected by microarray-based analysis for the corresponding mRNAs. Remarkably, AQP1 is generally assumed to be absent from the motor cortex, with a predominant expression in the brain in the choroid plexus [16] and in glial cells in the peripheral nervous system [17]. We therefore also performed immunofluorescence analyses of motor-cortical sections to elaborate our findings (Figure 6C), demonstrating the presence of AQP1 in non-neuronal cells in the motor cortex.

### Comparison with SALS spinal-cord and AD hippocampus expression data

We then compared expression ratios diseased vs. control for the SALS motor cortex to previously published data for spinal cord grey matter [4]. A comparison of all 2266 genes reliably detected in both tissues showed a negligible correlation (r = 0.04), while 23 of the motor-cortex candidate genes also present in the reduced data set showed a high correlation (r = 0.85) between both tissues (see the *Methods *section and Table 3).

**Table 3:** Comparison of expression ratios in motor cortex and spinal cord

		**Expression Ratios**		
**Gene Symbol**	**UniGene ID**	**Motor Cortex**	**Spinal Cord**	**Comparison**
AQP1	Hs.76152	2.52	2.84	==
MT2A	Hs.418241	2.43	4.66	=
SLC14A1	Hs.101307	2.15	1.91	==
ANXA2	Hs.511605	2.06	4.49	=
MT1X	Hs.374950	2.02	1.99	==
FCGBP	Hs.111732	2.00	3.14	=

DSCR1L1	Hs.440168	0.49	0.49	==
PCP4	Hs.80296	0.49	0.60	==
SCG5	Hs.156540	0.48	0.58	==
UCHL1	Hs.518731	0.48	0.48	==
GABRA1	Hs.175934	0.47	0.56	==
EGR1	Hs.326035	0.46	0.42	==
OLFM1	Hs.522484	0.43	0.50	==
VSNL1	Hs.444212	0.35	0.38	==

SNAP25	Hs.167317	0.31	0.82	<>
MDH1	Hs.526521	0.41	0.68	<>
CHN1	Hs.380138	0.42	0.71	<>
EIF4A2	Hs.478553	0.45	0.98	<>
RTN1	Hs.368626	0.46	0.71	<>
ATP5A1	Hs.298280	0.48	1.13	<>
NDUFA4	Hs.50098	0.48	0.73	<>
PCSK1	Hs.78977	0.39	1.73	<>
CHGB	Hs.516874	0.39	1.65	<>

Expression ratios for candidate genes identified in the present report (Motor Cortex) are compared to those found by Dangond et al. [4] (Spinal Cord), giving a correlation coefficient of r = 0.85. Spinal cord data were retrieved from the Gene Expression Omnibus Database (GEO, series GSE833) and processed as described in the *Methods *section.

== both tissues give the same result, differing ≤1.5-fold

= both tissues give the same result, differing >1.5-fold

<> both tissues give different results (up- vs. down-regulation or deregulation vs. constancy, defining constancy as a ≤1.5-fold deregulation)

To assess in how far responses detected in the motor cortex of SALS patients might be shared with other neurodegenerative diseases, we then compared our data to expression ratios diseased vs. healthy for previously published data by Blalock et al. [18] for the hippocampus of patients with severe Alzheimer's disease (AD). A comparison of all 6375 genes reliably detected in both tissues showed a low correlation (r = 0.38), while 38 of the motor-cortex candidate genes also present in the reduced data set showed a moderate correlation (r = 0.70) between both tissues (see the *Methods *section and Table 4).

**Table 4:** Comparison of expression ratios in patients with SALS and Alzheimer's disease

		**Expression Ratios**		
**Gene Symbol**	**UniGene ID**	**SALS**	**AD**	**Comparison**
AQP1	Hs.76152	2.52	1.62	=
CEBPD	Hs.440829	2.52	1.77	==
SERPINA3	Hs.534293	2.38	2.40	==
SLC14A1	Hs.101307	2.15	2.20	==

SLC12A5	Hs.21413	0.50	0.40	==
CYCS	Hs.437060	0.49	0.52	==
DSCR1L1	Hs.440168	0.49	0.39	==
NRGN	Hs.524116	0.49	0.41	==
PCP4	Hs.80296	0.49	0.32	=
ATP5A1	Hs.298280	0.48	0.61	==
CAP2	Hs.132902	0.48	0.52	==
GNAS	Hs.125898	0.48	0.48	==
NDUFA4	Hs.50098	0.48	0.62	==
UCHL1	Hs.518731	0.48	0.67	==
YWHAH	Hs.226755	0.48	0.53	==
PNMA2	Hs.521466	0.46	0.56	==
RTN1	Hs.368626	0.46	0.37	==
OLFM1	Hs.522484	0.43	0.55	==
RTN4	Hs.429581	0.43	0.75	=
CHN1	Hs.380138	0.42	0.50	==
MDH1	Hs.526521	0.41	0.54	==
BEX1	Hs.334370	0.40	0.63	=
NEF3	Hs.458657	0.38	0.28	==
VSNL1	Hs.444212	0.35	0.26	==
ENC1	Hs.104925	0.34	0.74	=
NEFL	Hs.521461	0.32	0.35	==
SNAP25	Hs.167317	0.31	0.59	=

MT2A	Hs.418241	2.43	1.31	<>
MT1E	Hs.534330	2.29	1.32	<>
MT1G	Hs.433391	2.19	1.36	<>
ANXA2	Hs.511605	2.06	1.45	<>
MT1X	Hs.374950	2.02	1.39	<>
ATP1A3	Hs.515427	0.49	1.46	<>
ATP6V1A	Hs.477155	0.49	0.78	<>
EGR1	Hs.326035	0.46	1.63	<>
EIF4A2	Hs.478553	0.45	0.75	<>
NSF	Hs.431279	0.45	1.01	<>
CHGB	Hs.516874	0.39	2.37	<>

Expression ratios for candidate genes identified in the present report (SALS) are compared to those found for data by Blalock et al. [18] for Alzheimer's disease (AD), giving a correlation coefficient of r = 0.696. AD data were retrieved from GEO (series GSE1297) and processed as described in the *Methods *section.

== both tissues give the same result, differing ≤1.5-fold

= both tissues give the same result, differing >1.5-fold

<> both tissues give different results (up- vs. down-regulation or deregulation vs. constancy, defining constancy as a ≤1.5-fold deregulation)

Despite the distinctive clinical manifestations of AD and its action on different areas of the central nervous system, therefore, global responses in the AD hippocampus correspond more closely to those in the SALS motor cortex than do those in the SALS spinal cord. This observation might reflect similar general changes in both brain tissues under long-term stress and after prolonged disease pathology, and ties in with the implication of processes such as oxidative stress, reduced energy metabolism, intracellular protein aggregation, and inflammation in both, AD and ALS pathogenesis. Importantly and similar to our findings for the SALS motor cortex, Blalock et al. [18] detected *ATP biosynthesis *and *glycolysis *by pathway-based analysis as significantly reduced in AD, and found deregulation in line with ours for *defense response *and *microtubule-based process*, albeit below the significance threshold. Changes significantly linked to the diseased state in our study, however, are highly correlated between SALS motor cortex and spinal cord, and less similar between SALS motor cortex and AD hippocampus. In particular, *ATP1A3*, *EGR1*, and *CHGB *experience opposing deregulation in the SALS motor cortex compared to the AD hippocampus, with *ANXA2*, *ATP6V1A*, *EIF4A2*, *NSF*, and all the metallothioneins detected in the hippocampus (*MT1E*, *MT1G*, *MT1X*, *MT2A*) also showing markedly different regulation between both tissues.

## Discussion

### A framework for the understanding of ALS pathogenesis

**Figure 7:**
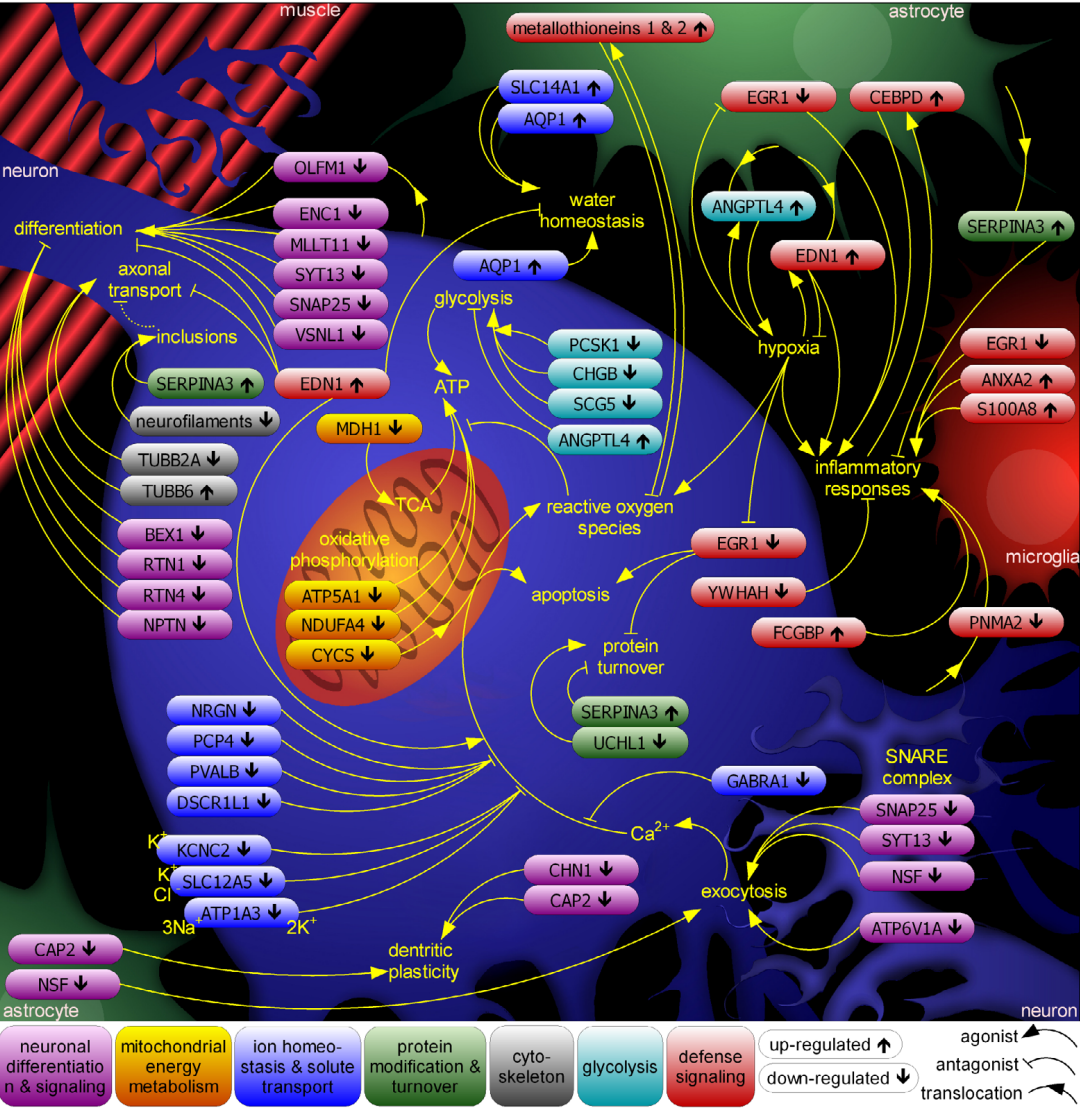
**The interplay of cell types and pathways in SALS pathology**. Combining pathways and candidate genes differentially expressed in the motor cortex of SALS patients reveals a complex interplay of cellular processes and cell types. Candidate genes corresponding to major pathways discussed in the main text are color-coded, placed in the cell type(s) where they most likely exert their influence, and their individual up- () or down-regulation () is indicated. Genes *EIF4A2*, *GNAS*, and *NAP1L5 *are left out for clarity.

A combination of candidate genes and pathway-based analysis provided a comprehensive view, at the level of mRNA, of the processes involved in ALS pathogenesis, suggesting an interpretation of changes in gene expression within a framework of deregulated pathways (Figure 7). It should be pointed out that, inherent to the analysis of end-stage diseased tissue, microarray analysis cannot distinguish between bona-fide up- and down-regulation of a gene and the selective survival and death, respectively, of cells highly expressing it. This ambiguity generally does not alter the interpretation of the role a given candidate gene with an established function might play in disease pathology. Moreover, selective cell death or survival appear unlikely explanations for the identification of candidate genes with two-fold changes of expression. However, this limitation needs to be borne in mind and will not be re-itterated in the course of the discussion. Nearly all candidate genes identified by this study may be actors in ALS pathogenesis based on their existing characterizations, and some of them stand out as potentially critical to de-regulated processes and to ALS therapy.

### Mitochondrial energy metabolism

*Mitochondrial energy metabolism *is a process down-regulated at the mRNA level and represented by all three major GO categories and by genes contributing to the tricarboxylic acid (TCA) cycle (*MDH1*) and involved in oxidative phosphorylation (*CYCS*, *NDUFA4 *and *ATP5A1*). The TCA accepts glycolysis-derived pyruvate as a key entry component. An alternative route for the supply of the TCA with pyruvate is its import downstream of the down-regulated MDH1, the cytosolic malate dehydrogenase [19]. Downstream of the TCA, oxidative phosphorylation occurs, for which we detected a specific down-regulation of genes encoding NDUFA4, a component of complex I of the electron transport chain in the inner mitochondrial membrane, CYCS, facilitating electron transport from complex III to complex IV in the intramembrane space, and ATP5A1, a component of the ATP synthase in the inner mitochondrial membrane. Importantly, the down-regulated CYCS is not only a component of mitochondrial oxidative phosphorylation, but also represents a cytoplasmic signal for neuronal apoptosis as a result of impaired mitochondrial integrity. Opening of the mitochondrial transition pore normally serves the ion exchange between matrix and cytosol, but is thought to be exaggerated upon excitotoxic levels of Ca^2+^, leading to release of CYCS from the mitochondrion [20]. CYCS release, its activation of cellular caspases, and subsequent apoptosis have been proposed as a contributing factor to neuronal cell death in ALS [1]. A lowered expression of CYCS, however, would conceivably decrease the sensitivity to stress-induced apoptosis [21]. More significantly, our observations expand a role for mitochondrial dysfunction in the death of ALS motoneurons, which is otherwise mainly understood as a consequence of mitochondrial recruitment of mutant SOD1 in FALS [1], and which was even ruled out for SALS by one study [22]. Importantly, the significant down-regulation of GO nodes relating to mitochondria can not be the mere consequence of mitochondrial dysfunction or reduced numbers of mitochondria, as the GO hierarchy does not represent mitochondrion-encoded transcripts. Moreover, nuclear-encoded transcripts encoding mitochondrial proteins are universally induced during conditions of oxidative stress and mitochondrial damage [23], contrary to our observations for motor-cortical cells of SALS patients.

### Glycolysis

*Glycolysis *was detected as significantly down-regulated in the SALS motor cortex by pathway-based analysis of our microarray data. Glycolysis represents the cytoplasmic alternative to mitochondrial ATP generation by the TCA cycle and by oxidative phosphorylation, but is also a major source of pyruvate supply to the TCA cycle. At the level of individual genes, significant changes in gene expression similarly indicate a reduction of glycolysis (*ANGPTL4*) and glucose availability (*CHGB*, *SCG5*, *PCSK1*). The up-regulation of *ANGPTL4 *reduces expression of genes involved in glycolysis and gluconeogenesis [24]. The down-regulation of granins CHGB and SCG5, both processed by the down-regulated prohormone convertase PCSK1, increases insulin levels, hence reducing the availability of glucose for glycolysis [25]. In the context of ALS-related oxidative stress and neuronal modulation of ion gradients, a reduction of glycolytic energy supply, in combination with reduced mitochondrial energy supply, would constitute a major cause for neuronal cell death.

### Ion homeostasis and solute transport

*Ion homeostasis and solute transport *is detected as reduced at the mRNA level by pathway-based analysis and covers both cytoplasmic and mitochondrial events (see Table 2). For instance, 71.7% of *monovalent inorganic cation transporter activity *includes genes involved in proton transport (*hydrogen ion transporter activity*, z = 13.444, *P *= 0), commonly associated with organelle membranes (e.g. of mitochondria and lysosomes), and genes involved in the transport of sodium ions (*sodium ion transporter activity*, z = 10.059, *P *= 0) and metal ions in general (*metal ion transporter activity*, z = 8.147, *P *= 0), more commonly associated with the plasma membrane. *Ion homeostasis and solute transport *comprises three groups of candidate genes relating to *excitotoxicity*, *calcium homeostasis*, and *water and solute transport*, respectively.

*Excitotoxicity*, the excessive signaling of glutamate receptors, is a key pathogenic mechanism proposed in ALS, and is aggravated by a weakening of inhibitory synaptic events mediated by the GABAA receptor. Alterations of its composition, such as the depletion of its GABRA1 subunit found in this study, was previously demonstrated in human motor cortex of ALS patients and is associated with heightened excitability [26]. Increased excitability, and hence exacerbated excitotoxicity, may also derive from the reduced expression of *ATP1A3*, *KCNC2*, and *SLC12A5*. Mutations of the plasma-membrane Na^+^/K^+ ^pump ATP1A3 have been linked to rapid-onset dystonia-parkinsonism [27]. Moreover, ATP1A3 expression is reduced in SOD1(G93A) FALS mice [28] and in motoneurons of Wobbler mice, a genetic model of spinal-cord motoneuronal degeneration, with progesterone treatment restoring both, *ATP1A3 *mRNA expression and viability of motoneurons [29]. KCNC2 regulates the voltage-dependent potassium ion permeability of excitable membranes, and its decreased expression may lead to decreased potassium conductance and hence delayed repolarization of axons. While KCNC2 itself is poorly characterized, defects in other voltage-gated potassium channels can lead to hyperexcitability and hypersensitivity to oxidative stress [30]. *SLC12A5 *encodes the neuronal isoform of the potassium-chloride cotransporter family. During early development, increased expression of *SLC12A5 *lowers the intraneuronal chloride concentration below its electrochemical equilibrium and allows GABA to act as an inhibitory neurotransmitter. Conversely, a switch of GABA action from inhibitory to excitatory has been proposed as a mechanism contributing to excitotoxicity in injured neurons. Indeed, down-regulation of *SLC12A5 *expression together with GABAA receptor-mediated excitation occurs after *in-vivo *axonal injury [31], and mouse *SLC12A5 *knockouts suffer severe motor deficits and immediate postnatal death by asphyxiation [32].

*Calcium homeostasis *and calcium-binding proteins are critical to preventing motoneuron death in ALS, and with *PVALB*, *DSCR1L1*, *NRGN*, and *PCP4 *we found four genes down-regulated in the ALS motor cortex that moderate the impact of intracellular calcium release. There is disagreement whether the calcium buffer PVALB represents a marker of motoneurons resistant to ALS-related cell death [33,34], but its reduction would likely increase cell death at the elevated calcium concentration typical of ALS [35]. Similarly, DSCR1L1 is an inhibitor of calcineurin [36], a mediator of many cellular responses, including apoptosis [37]. Depletion of the two calmodulin-binding proteins NRGN and PCP4, involved in moderating calmodulin-mediated calcium signaling, would further reduce resistance to calcium-mediated toxicity [38,39].

*Water and solute transport *is represented by the up-regulation of *AQP1 *and *SLC14A1*. Both, *AQP1 *and *SLC14A1*, experience up-regulation with age and by hypertonicity [40], and like AQP1 the urea transporter SLC14A1 may also function as an efficient water transporter [41]. Their joint up-regulation found in this study is indicative of disturbed ion and water homeostasis in the motor cortex of SALS patients. The water transporter AQP1 in particular is of interest through its association with synaptic vesicles and participation in their swelling in cells outside the central nervous system [42].

### Protein modification and turnover

*Protein modification and turnover *are essential to motoneuron survival, resulting in the selective death of motoneurons upon chemical inhibition of the proteasome complex [43], which we detected as down-regulated in SALS samples by pathway-based analysis of our microarray data, thus stressing a role of disturbed protein turnover in ALS pathogenesis. Indeed, the progressive accumulation of polyubiquitinated protein conjugates, indicating a failure of proteasomal turnover, is a consistent feature of many neurodegenerative diseases [44], including ALS [1]. In line with this we also observed the down-regulation of UCHL1, a neuron-specific ubiquitin carboxy-terminal hydrolase-1, which also displays a secondary dimerization-dependent ubiquitin-ligase activity and represents up to 2% of total soluble brain protein [45]. A point mutation affecting the ligase activity of UCHL1 has been linked to Parkinson's disease [46], while an internal 42-amino-acid deletion in UCHL1 is responsible for murine gracile axonal dystrophy, which is characterized by axonal degeneration and formation of spheroid bodies in nerve terminals [47]. The latter phenotype and the observation of poly-ubiquitinated aggregates in ALS create a tantalizing link between the hydrolase activity of UCHL1 and the depletion of *UCHL1 *mRNA in the motor cortex of SALS patients. Up-regulation of SERPINA3, a serine protease inhibitor enriched in neurofilament conglomerates of ALS motoneurons [48], can be seen in the same light and has been associated with other neurodegenerative diseases. In Alzheimer's disease, for instance, its astrocytic expression is elevated [49] and exacerbates the accumulation of protein aggregates [50]. SERPINA3 also has other effects, however, including protection against microglia [51].

### Cytoskeleton

The *cytoskeleton *is critical for neuronal maintenance and plasticity, neurite outgrowth, axonal caliber and transport. Our analysis reveals the alteration of two major components of the neuronal cytoskeleton in the ALS motor cortex at the mRNA level, showing a general down-regulation of microtubules by pathway-based analysis and deregulation of two genes encoding tubulin beta (*TUBB2A*, *TUBB6*), as well as decreased expression of all three neurofilament subunits (NEFL, NEF3, NEFH). A depletion of microtubules and neurofilaments has deleterious effects on motoneurons, according to our present understanding of their role in ALS pathogenesis [1]. Impaired microtubule-based axonal transport causes related motoneuropathies and is the earliest detectable, presymptomatic abnormality in *SOD1*-mutant FALS mice. What is more, defects in microtubule-associated motor proteins cause ALS-related human motoneuropathies, such as Charcot-Marie-Tooth disease 2A [52] and hereditary spastic paraplegia [53], and are responsible for ALS phenotypes in Drosophila [54] and mouse [55]. The role of neurofilaments (NFs) in ALS is still controversial, despite a substantial body of research addressing the subject [1]. NF assemblies have been suggested as a possible impediment to axonal transport, either as its aberrantly crosslinked cargoes or in stationary aggregates, and even disturbing the stoichiometric balance between NF subunits can lead to ALS-like morphology and death of motoneurons. However, despite a raised incidence of mutations in *NF *genes in ALS subjects, a comprehensive study has excluded mutations in *NF *genes as a primary cause for ALS [56]. Moreover, ALS pathogenesis also progresses without axonal neurofilaments [57], and over-expression of NEFH prolongs survival of *SOD1*-transgenic FALS mice, prompting the hypothesis that NFs primarily act as an abundant buffer for otherwise deleterious processes, such as actions of *SOD1 *gain-of-function mutants [58] or aberrant tyrosine nitrosylation or phosphorylation [1,59,60]. The down-regulation of all three NF subunits observed in this study would be detrimental according to either interpretation of NF involvement in ALS pathology, first by interfering with their stoichiometric balance and second by depleting their availability as a potential buffer for aberrant enzymatic activities.

### Neuronal maintenance and signaling

Changes in *Neuronal maintenance and signaling*, detected by gene-based analysis of our microarray data, comprise only down-regulated genes and tie in with observations for disturbed signaling in the motor cortex of sporadic, non-SOD1-linked ALS [12,13]. Depleted are general promoters of neurodifferentiation and axonal and dendritic maintenance (*CAP2*, *ENC1*, *MLLT11*, *OLFM1*, *VSNL1*), with clear implications for neuronal survival, but also several inhibitors of differentiation and promoters of cell proliferation (*BEX1*, *CHN1*, *RTN1*, *RTN4*, *NPTN*), whose role in ALS pathogenesis is ambiguous. Four additional genes (*ATP6V1A*, *NSF*, *SNAP25*, *SYT13*) encode proteins regulating synaptic vesicle processing and exocytosis, processes highly relevant to ALS pathology [1]. While most of the above genes are known to be expressed in multiple cell types, some of them (*CHN1*, *NPTN*, *SNAP25*, *OLFM1*, *VSNL1*) are neuron-specific. Importantly, we had ensured selecting patient material that exhibited good histological integrity of the motor cortex (see Figure 1), thus minimizing the possibility that observations for neuron-specific genes would reflect the loss of motoneurons typical of ALS.

*MLLT11 *expression is up-regulated in differentiated cortical neurons, and its ectopic expression in embryonic kidney cells triggers the expression of neuronal markers [61]. OLFM1, a secreted glycoprotein, is also strongly expressed in cerebral cortex [62,63] and has been implicated in neural crest development in birds [64] and neurogenesis in *Xenopus *[65]. ENC1 is a matrix protein expressed almost exclusively in the brain, whose overexpression induces neuronal process formation and whose knock-down inhibits neurite development [66]. Similarly, the calcium-dependent action of VSNL1 has been linked to the promotion of axonal differentiation and to axonal plasticity [67]. The multifunctional protein encoded by *CAP2 *links the actin cytoskeleton and cAMP signaling, and might therefore facilitate intracellular signal transduction events, dendritic plasticity and synaptic membrane recycling [68,69].

Amongst the down-regulated inhibitors of neurodifferentiation in the SALS motor cortex are BEX1, a promoter of cell proliferation [70], and RTN1 and RTN4, two members of the reticulon family thought to play an important role in the regulation of neuronal regeneration. RTN4, in particular, is a potent neurite outgrowth inhibitor and has been shown to inhibit the regeneration of severed axons in the mammalian central nervous system [71]. *NPTN *encodes a protein involved in cell-cell interactions that inhibits neurite outgrowth from dorsal root ganglion and forebrain neurons [72]. Finally, *CHN1 *encodes the Rac GTPase activating protein alpha-1-chimerin, which mediates the pruning of dendrites in response to synaptic activity [73].

ATP6V1A is a subunit of the vacuolar proton pump V-ATPase, which generates a proton gradient across the membrane of maturing synaptic vesicles, required for the accumulation of neurotransmitters within them [74]. SNAP25 is a mediator of axonal repair, a regulator of calcium signaling, and a component of the NSF-binding SNARE complex, essential to vesicle fusion and recycling [75], and therefore has an impact on at least three processes critical to ALS pathogenesis. Like alsin, a likely regulator of endosome dynamics and a causative gene for juvenile forms of ALS [1], SNAP25 is also involved in endosome recycling [76], creating a fourth, albeit tentative, link of SNAP25 to ALS pathogenesis. SYT13, like SNAP25, participates in neurite outgrowth and axonal repair, and unlike usual synaptotagmins binds to cellular membranes in a Ca^2+^-independent fashion to facilitate membrane fusion and exocytosis [77]. It is unclear whether SYT13, like other synaptotagmins, does so by interaction with SNAP25. Following membrane fusions the down-regulated NSF, a key component of internal and exocytotic vesicle transport [78], facilitates the ATP-dependent recycling of SNARE proteins, such as SNAP25.

### Defense signaling

*Defense signaling *has been implicated in ALS pathogenesis by numerous studies showing inflammatory and immune responses [1], and linking the detection of IgG in ALS motoneurons to neuronal apoptosis [79]. Additional evidence is provided by the detection of IgG in motoneurons of the spinal cord and in pyramidal cells within the motor cortex of ALS patients [80-82] and by the ability of IgG from patients with ALS to induce apoptotic neuronal cell death [79,83,84]. Importantly, IgG purified from ALS patients was found to induce synaptic transmission through interaction with presynaptic membranes, relating autoantibodies in ALS to excitotoxicity [85]. In the present study defense signaling comprises the only up-regulated GO categories in the SALS motor cortex, and is represented by numerous candidate genes, whose differential expression likely promotes (*FCGBP*, *YWHAH*, *ANXA2*, *S100A8*, *EDN1*, *CEBPD*) or suppresses (*EGR1*, metallothioneins) inflammatory responses, respectively. Down-regulation of *PNMA2*, whose product is a target of neurodegenerative autoimmune responses, is reminiscent of a PNMA2-autoantibody-associated encephalitis patient with ALS symptoms, who presented with inflammation in cortex and spinal-cord grey matter [86,87]. With a suspected contribution of inflammatory responses to ALS pathogenesis, this suggests the targeting of PNMA2 by neurodegenerative autoimmune responses, leading to a depletion of PNMA2-positive cells. In line with this, the up-regulated *FCGBP*, encoding an IgG-Fc binding protein, may be involved in immune protection and inflammation [88], and shows elevated serum levels in patients with autoimmune disorders [89]. Elevated levels of FCGBP could therefore contribute to ALS pathogenesis by facilitating autoimmune and neuroinflammatory responses. Up-regulation of *CEBPD*, encoding a transcription factor of cytokines and other pro-inflammatory response genes, elevated in the brain of Alzheimer's disease patients and itself induced by inflammatory stimuli, ties in with this assumption [90]. Similarly, the down-regulation of *YWHAH*, a gene linked to schizophrenia that encodes a transcriptional activator of the glucocorticoid receptor [91] and therefore of anti-inflammatory responses, can also be predicted to aggravate neuroinflammation. The up-regulated candidate genes *ANXA2 *and *S100A8 *encode two Ca^2+^-modulated proteins expressed in microglia and are implicated in the intracellular and extracellular regulation of inflammatory responses [92,93]. ANXA2 promotes generation of plasmin on the cell surface [94], a process linked to excitotoxin-induced neurodegeneration [95]. S100A8 mediates inflammatory responses, such as the induction of chemokines, and has been linked to the reduction of cell junction proteins and of membrane integrity [96].

The multifunctionality of EDN1 and EGR1 make the consequences of their differential expression in the SALS motor cortex ambiguous. For instance, the up-regulated *EDN1*, encoding a pro-inflammatory vasoconstrictor peptide, exerts a variety of ALS-aggravating effects, including axonal degeneration [97], impairment of water homeostasis, heightened sensitivity to hypoxic stress [98], and increased excitotoxicity [99]. However, neuron-specific *EDN1*-knockout mice show elevated sensitivity to pain [100], and EDN1 expression in astrocytes is required for their survival under oxidative stress [101]. Moreover, the hypoxia-inducible EDN1 promotes angiogenesis, reducing the possible effects of hypoxia-related oxidative stress [101,102], amongst other mechanisms by activation of the vascular endothelial growth factor. On the other hand, down-regulation of EGR1, a master switch of inflammatory responses prompted by oxidative stress [103], counteracts angiogenesis, thus increasing hypoxic conditions, but has an anti-inflammatory effect. It is important to note that the up-regulated ANGPTL4 (see **Glycolysis **above) and the down-regulated EGR1, two inducers of angiogenesis [6,104], represent a positive and a negative target of the hypoxia-induced peroxisome-proliferator-activated receptor gamma [103,105], respectively. Therefore the differential expression of *EGR1*, *ANGPTL4*, and *EDN1 *indicates hypoxic conditions in the SALS motor cortex, tying in with a predisposition for ALS by mutation of angiogenin in man [106] and of vascular endothelial growth factor in mouse [107].

Indicating protective responses in the SALS motor cortex, we observed the up-regulation at the mRNA level of metallothioneins (MT1E, MT1G, MT1L, MT1M, MT1X, MT2A), neuroprotective, anti-oxidant stress-response proteins induced by metal ions, cytokines, and other stress stimuli [108]. Metallothioneins of the *MT1 *and *MT2 *families are expressed in astrocytes, and are increased in spinal cord of ALS patients and in transgenic mutant-*SOD1 *mice, where their experimental reduction significantly reduces survival. Importantly, endogenous *MT1 *and *MT2 *are undetectable in pure motoneuron cultures, which can be protected against oxidative stress by experimental *MT1 *overexpression [109], indicating the importance of metallothionein-mediated protection normally provided by astrocytes.

### Orphan genes

*Orphan genes*, candidate genes with wide-ranging functions (*EIF4A2*) or without sufficient functional characterization (*NAP1L5*, *GNAS*), can only be associated tentatively with major deregulated pathways identified in this study, and were omitted from Figure 7. The down-regulated translation initiation factor EIF4A2 is interchangeable with EIF4A1 for the initiation of translation in general, and induces neural folding during embryonic development [110]. Interestingly, *EIF4A2 *has also been linked to type-2 diabetes and in rat cells appears to suppress insulin expression [111]. Its down-regulation, like that of *CHGB*, *SCG5*, and *PCSK1*, would therefore lead to elevated insulin levels and hence reduced glucose availability (see **Glycolysis **above). The down-regulated *NAP1L5 *is an imprinted and largely uncharacterized gene, highly expressed in brain [112]. NAP1L5 has sequence similarity to NAP1, a cytoplasmic-nuclear shuttle protein for histones, that has large-scale effects on gene expression by chromatin restructuring [113]. The *GNAS *gene is also down-regulated and, like *NAP1L5*, is imprinted [114]. Alternative first exons for *GNAS *transcripts lead to distinct products, which in human have been linked to osteodystrophy and pseudohypoparathyroidism phenotypes, while in mouse *GNAS *heterozygous mutants show metabolic abnormalities and insulin hypersensitivity (see **Glycolysis **above).

## Conclusion

### Global reduction of energy supply

Pathway-based and gene-based analyses show a general down-regulation of nuclear genes encoding mitochondrial components in the motor cortex of ALS patients. Although at first glance tying in with ALS-related mitochondrial dysfunction, this observation is at odds with the heightened level of oxidative stress typical of ALS. Numerous studies have characterized oxidative stress as a general inducer of nuclear genes that boost replication, maintenance and repair of mitochondria, as compensatory action for stress-related mitochondrial damage and death (see [23]). The down-regulation observed in the present study suggests alternative signaling to achieve a targeted depletion of mitochondrial components in the SALS motor cortex, which might therefore be a cause rather than a consequence of ALS pathology.

As for mitochondrial energy supply, pathway-based and gene-based analyses have independently indicated a reduction in glycolytic activity. Experimental inhibition of glycolysis in an FALS cell-culture model diminishes cell viability, suggesting that ALS-related mitochondrial dysfunction critically reduces cellular ATP supply [115], while hypoglycemia is a strong inducer of oxidative stress, even in the absence of such aggravating factors [116]. Now our results indicate that reduction in glycolytic energy supply is in itself a factor contributing to ALS pathology, suggesting as one overriding cause of ALS-related neuronal cell death the depletion of intracellular ATP levels by a combined reduction of the TCA cycle, oxidative phosphorylation and glycolysis.

Reduced intracellular ATP levels lead to increased susceptibility to cell death upon oxidative [117] and excitotoxic [118] stress, and would be particularly detrimental to the survival of neurons, as the re-establishment of Ca^2+ ^gradients and of general ion homeostasis following neuronal signaling is highly ATP-dependent. As an aggravating factor, coordinated Ca^2+ ^signaling is facilitated in neurons by strategic positioning of mitochondria near endoplasmic-reticulum Ca^2+^-release sites [20]. Therefore, reduced availability of ATP would lead to a downward spiral of an inability to remove intracellular Ca^2+^, Ca^2+^-induced mitochondrial production of reactive oxygen species, and additional depletion of mitochondrial ATP supply.

### The importance of addressing all cell types in ALS therapy

During our expression analysis of the motor cortex we identified several candidate genes, such as *AQP1*, *CEBPD*, *SERPINA3*, and the *MT1 *and *MT2 *family members, which are exclusively expressed in non-neuronal cells. Particularly in the analysis of excitotoxicity and defense signaling, involving astrocytes and microglia, and in the interplay of inflammatory versus anti-inflammatory and of cell-death versus cell-rescue responses, the importance of different cell types involved in ALS pathology becomes apparent. The survival of motoneurons is highly dependent on their cellular environment, which is to a large majority made up of non-neuronal cells [119]. Data on the interaction of neurons with glial cells in the context of ALS are still patchy, but there is a growing understanding that non-neuronal cells have a fundamental role to play in the pathogenesis of neurodegenerative diseases [119]. Our data indicate a general increase of microglial activity and immune responses, likely aggravating ALS disease symptoms. It is known, however, that the role of microglia in neuropathies extends beyond immune responses alone. Only recently, activated microglia have been recognized to hypersensitize sensory neurons, and their role in motoneuronal excitotoxicity still remains to be investigated [120]. Moreover, our data show mixed signals from astrocytes, comprising inflammatory and anti-inflammatory responses, which have to be evaluated on an individual basis and might be modulated to slow disease progression. Astrocytes might have significant impact on the excitotoxicity and the oxidative stress responses detected by our study, as they are in close interaction with motoneurons and neuronal synapses, and themselves express all the components required for Ca^2+^-responsive vesicular glutamate release [121,122].

### Perspectives for ALS therapy

In the absence of effective treatments for ALS, the search for potential therapeutics continues. Like Riluzole, the only approved ALS treatment to date, the vast majority of drugs currently considered for clinical trials are based on animal models with SOD1-mutant background (85% in a recent survey [123]). Only an estimated 2% of ALS cases, however, are linked to SOD1, and resulting treatments might therefore, similar to Riluzole, have limited value as general ALS therapies. However, several candidate compounds for ALS therapy [123] address aspects of SOD1 mutants, such as oxidative stress, impaired metal binding, and defects in protein folding, that we have also identified as affected in the motor cortex of sporadic, non-SOD1-linked patients.

A successful general therapy of ALS will have to consider the interplay and individual roles of different pathways and cell types in pathogenesis. The comprehensive analysis described here provides a full molecular portrait of the changes occurring in the motor cortex of SALS patients and provides new leads for the development of effective ALS therapies. Indeed, for the manipulation of many of the genes and pathways implicated by this study, experimental or therapeutic drugs, not yet discussed in the context of ALS, are already available [124,125]. Moreover, our findings suggest that several drugs, currently used to treat unrelated diseases, might be of benefit to ALS patients. These include bosentan, an EDN1-receptor antagonist employed against diabetes and pulmonary hypertension [126]. The widely used anti-inflammatory drug ibuprofen is an antagonist of isoleucin-1β and of its downstream target SERPINA3 [127]. GABA antagonists such as zopiclone, used to treat insomnia, are of potential benefit to GABRA1-related excitotoxicity [128]. Significantly, symptom progression was stopped in a patient presenting with anti-PNMA2-related motoneuron degeneration by a combination of corticosteroids, intravenous immunoglobulin and antiepileptic drugs [86]. Similarly, and although it is clear from *SOD1*-linked FALS that all the alterations detected in ALS can have a common root and are therefore interrelated, therapy of ALS might also require combined medication to combat disease progression at multiple fronts. Many of the potential targets of this battle have been outlined by this study.

## Methods

### Characteristics of subjects

Two groups of patient samples, consisting of eleven SALS and nine control subjects, were used in this study. Fresh-frozen samples of human motor cortex (precentral-gyrus sections) were obtained from the NICHD Brain and Tissue Bank for Developmental Disorders under contracts NO1-HD-4-3368 and NO1-HD-4-3383 and selected for post-mortem intervals (PMI) prior to freezing not exceeding 24 hours. Additionally, two patient samples were obtained as total RNA from a commercial source (Ambion, Inc.). All sample material is certified to have been obtained following international ethical guidelines and with prior consent from a fully informed donor or a member of the donor's family. All disease samples were cases of spontaneous ALS, with a mean patient age of 68.2 ± 7.6 years. Control samples had a mean patient age of 68.7 ± 11.0 years and were selected for matching age and for causes of death unrelated to ALS or other neurological disorders. Detailed information related to source code, age, sex, race, and storage of patient samples is given in Table 1.

### Sample preparation

Individual slices of 10 μm were produced from tissue samples at -20°C by a Leica CM1510S cryostat (Leica Microsystems) and stored at -80°C until further processing. Two slices per sample were stained by hematoxylin/eosin staining (Bio Optica) and for Nissl substance (with a microfiltered solution of cresyl violet, Sigma), respectively, to assess integrity of cellular and tissue morphology. Individual slices were used for *in-situ *hybridization and immunofluorescence (see below). Ten adjacent slices per sample were pooled and used for RNA extraction with Trizol (Invitrogen) following the manufacturer's standard protocol, followed by confirmation of RNA integrity by agarose gel electrophoresis.

### Microarray processing and data extraction

Complementary RNAs (cRNAs) labeled with Cy5-CTP (Perkin-Elmer) were synthesized from 1 μg of total RNA of each sample using the Low RNA Input Fluorescent Linear Amplification Kit (Agilent Technologies) following the manufacturer's protocol. A reference cRNA, labeled with Cy3-CTP (Perkin-Elmer), was synthesized from 1 μg of sample D2 RNA. Aliquots (750 ng) of Cy3 and Cy5 labeled cRNA targets were co-hybridized on Whole Human Genome Oligo Microarrays (Agilent Technologies). Microarray hybridization and washing were performed using reagents and instruments (hybridization chambers and rotating oven) as indicated by the manufacturer (Agilent Technologies).

Microarrays were scanned at 10-μm resolution using a GenePix Personal 4100A microarray scanner and the GenePix Pro 6.0 acquisition and data-extraction software (Molecular Devices, Corp.). Raw data were processed and analyzed with Acuity 4.0 (Molecular Devices, Corp.) and GeneSpring 7.2 (Agilent Technologies). To remove unreliable data, all genes from all samples were filtered for quality to include only probe data fulfilling all of the following criteria: the spot had < 3% of saturated pixels at 635 and 532 nm; the spot was not flagged "bad", "not found" or "absent"; the spot had relatively uniform intensity and uniform background (Rgn R^2 ^635/532 > 0.6); the spot was detectable well above background (signal-to-noise ratios at 635 or 532 nm > 3). Filtering data by quality control criteria short-listed 19,431 genes as our complete data set for Lowess normalization and subsequent analyses, out of a total of 44,233 probes (including control and alignment probes) present on the microarrays. The microarray data have been deposited in the Gene Expression Omnibus (GEO) database [129] under accession no. GSE4595.

### Pathway-based microarray analysis

To analyze the gene expression changes in the context of known biological pathways we used the Gene Map Annotator and Pathway Profiler (GenMAPP) 2.0 software package [130].

Input data for GenMAPP analysis were unique probe identifiers, corresponding UniGene cluster IDs, an average fold-change and an uncorrected *P *value (Welch's t-test) diseased vs. control for all 19,431 short-listed genes of our complete data set. If a UniGene cluster ID was represented by multiple probes, the probe carrying the lowest (most significant) uncorrected *P *value was used for pathway-based comparison with spinal-cord data [4] (see below). GenMAPP dynamically links gene-expression data to the gene ontology (GO) hierarchy of biological processes, cellular components and molecular functions [131]. For each of the GO categories in the hierarchy (nodes), and including all the genes in its child nodes, GenMAPP identifies genes that meet a user-defined criterion (≥ 1.2-fold-change in our analysis, as recommended by [132] and [133]). From the total number of genes analyzed, the number of genes in a category and the corresponding numbers of genes meeting the queried criterion a z score is calculated, which indicates the non-randomness (for high, positive z scores) of the proportion of genes meeting the criterion. Additionally, a corrected *P *value is calculated through permutation analysis (2000 permutations) of the data, followed by Westfall-Young adjustment for multiple hypothesis testing. Permutation analysis sets limits to the resolution of extremely significant events, so that the *P *value for all events with an uncorrected *P *< 0.0005 equals zero. Only nodes with positive z scores and a corrected *P *< 0.05 were considered in our analysis, with a higher z score indicating greater significance between nodes of identical *P *value.

### Gene-based microarray analysis

Of our quality-filtered data set of 19,431 genes, those with an average change greater than twofold were screened by a two-sided one-way ANOVA using Welch's t-test (with a Kolmogorov-Smirnov-test *P *value of 0.38 or above indicating a normal distribution of tested values), followed by the Benjamini and Hochberg False Discovery Rate procedure as a multiple testing correction. Genes with a corrected *P *value < 0.05 were selected as differentially expressed candidate genes. All candidate probe sequences were tested against the NCBI nucleotide database (November 2005) by BLASTN to update their annotations and confirm the specificity of each probe.

### Quantitative RT-PCR

Sample total RNA (2 μg) was reverse transcribed with the Protoscript reverse transcription kit (New England Biolabs) using the VN(dT)_23 _primer as recommended by the manufacturer. As a standard for relative RNA quantification (Standard cDNA), ten equivalent reactions using 2 μg pooled RNA from healthy control samples (222 ng each) were performed and the resulting cDNAs precipitated and resuspended in 50 μl water each. As a negative control for cDNA quantification, a further reaction using 2 μg of pooled RNA was performed, in which the reverse transcriptase was replaced by water. All cDNAs were tested by conventional PCR with primers for glyceraldehyde-3-phosphate dehydrogenase (New England Biolabs), using 1/50 of each cDNA (the equivalent of 40 ng total RNA).

Quantitative RT-PCR (qRT-PCR) amplifications were performed with a LightCycler (Roche Molecular Biochemicals) using the same starting amount and LightCycler^® ^FastStart DNA MasterPLUS SYBR Green I reagents in a standard volume of 20 μl. Real-time detection of fluorimetric intensity of SYBR Green I, indicating the amount of PCR product formed, was measured at the end of each elongation phase. Fluorescence values measured in the log-linear phase of amplification were considered using the second-derivative-maximum method of the LightCycler Data Analysis software (Roche Molecular Biochemicals). Relative quantification was performed using serial dilutions of pooled Standard cDNA (the equivalent of 80, 40, 20, 10 and 5 ng total RNA, respectively, run in duplicate) to provide a standard curve for each run. For all experiments, the standard curve had an error of below 5% and extended over the relative quantities of all individual samples.

Candidate genes whose differential expression was tested by gene-specific qRT-PCR analysis were parvalbumin and the metallothioneins *MT1B*, *MT1E*, *MT1G*, *MT1L*, *MT1M*, *MT1X *and *MT2A*. Differences in the quantity of starting material were compensated by normalization with the housekeeping genes beta-2-microglobulin (B2M) and ribosomal protein L19 (RPL19). The primers used are detailed below. Normalized fold-changes between diseased and healthy samples were calculated and tested by a two-tailed Mann-Whitney U-test (not assuming equal variances).

### *In-situ *hybridization

cDNAs corresponding to four mRNAs were synthesized by PCR from human brain RNA using specific forward and reverse primers. PCR fragments were subcloned into pCR4Blunt-TOPO vector (Invitrogen) and the orientation of the insert was determined by sequencing. Following linearization of plasmids by restriction endonucleases, riboprobes containing Cy3-CTP (sense) or Cy5-CTP (antisense) (Perkin-Elmer) were synthesized by T3 and T7 polymerase, respectively, using the MAXIscript In Vitro Transcription Kit (Ambion, Inc.). The *in-situ *hybridization procedure was performed as previously described [134]. Fluorescent hybridization signals were obtained by scanning sections at 5-μm resolution using a GenePix Personal 4100A scanner (Molecular Devices, Corp.). No signal was detected in control brain sections hybridized with the sense riboprobes or pretreated with RNase before hybridization with the antisense probes. Evaluation of hybridization signals were obtained by using a computer-assisted image analysis system and the Photoshop 7.0 software (Adobe Systems, Inc.).

### Immunoblots

Protein was extracted from human motor-cortex samples using standard methods. Following SDS-PAGE separation on 10–12% gels and semi-dry protein blotting (transfer buffer 10 mM NaHCO_3_, 3 mM Na_2_CO_3_, 20% methanol, pH 9.9) to nitrocellulose Hybond membranes (Amersham Pharmacia Biotech), blots were blocked (0.1% Tween, 3% milk PBS), incubated with the appropriate primary antibody at 4°C over-night, and following three washes with PBS were incubated with secondary antibody. Primary antibodies (all Santa Cruz Biotechnology, Inc.) were rabbit antibodies ANXA2 (SC-9061), AQP1 (SC-20810), and CANX (SC-11397), and goat antibody NRGN (SC-18336), all at 1:100 dilution. As HRP-conjugated secondary antibodies (all Amersham Pharmacia Biotech) we applied a 1:4000 dilution of anti-rabbit antibody for detection of ANXA2, AQP1, and CANX, and of anti-goat antibody for NRGN. Immunoblots were visualized using the Enhanced Chemiluminescence System (Amersham Pharmacia Biotech). Band intensities were determined as background-corrected volume measurements using the ImageQuant TL software (Amersham Pharmacia Biotech), were equalized using CANX as a loading reference (which has not been linked to ALS and in our microarray analysis showed an mRNA ratio diseased vs. control of 0.97), and were subjected to statistical analysis using a two-tailed, heteroscedastic Student's t-test in Excel (Microsoft Corp.).

### Primers

The following forward (FP) and reverse (RP) primers were used for qRT-PCR analysis: *PVALB *(GenBank Accession No: NM_002854, forward primer (FP): 5'-acgctgaggacatcaagaagg-3', 5'-caattttgccgtccccatc-3), reverse primer (RP): *MT1B *(GenBank Accession No.: NM_005947, FP1: 5'-actccaggcttgtcttggctcc-3', RP1: 5'-tgggagcagggctctcccaa-3', FP2: 5'-ttgcctaggaactccaggcttgt-3', RP2: 5'-gcagcggcacttctctgatgag-3', FP3: 5'-tgctgctcttgctgccccgt-3', RP3: 5'-aaagaatgtagcaaaccggtcaggg-3'), *MT1E *(GenBank Accession No.: NM_175617, FP: 5'-ccttcttccccaggctgctgt-3', RP: 5'-aatgcagcaaatggctcagtgttg-3'), *MT1G *(GenBank Accession No.: BC035287, FP: 5'-gcatctgcaaaggggcatcg-3', RP: 5'-aaaggaatgtagcaaaggggtcaaga-3'), *MT1L *(GenBank Accession No.: BC070351, FP: 5'-gggctcctgctcctgtgcca-3', RP: 5'-ggaatgtagcaaatgctcagggttg-3'), *MT1M *(GenBank Accession No.: NM_176870, FP: 5'-tggtgtctcctgcgcctgca-3', RP: 5'-aatgcagcaaatggctcagtatcgtatt-3'), *MT1X *(GenBank Accession No.: BC053882, FP: 5'-tgctgctcctgctgccctgt-3', RP: 5'-aaaagatgtagcaaacgggtcaggg-3'), *MT2A *(GenBank Accession No.: NM_005953, FP: 5'-cgactctagccgcctcttca-3', RP: 5'-gaaaaaggaatatagcaaacggtcac-3'), *RPL19 *(GenBank Accession No.: NM_000981, FP: 5'-ggctgctcagaagataccgtg-3', RP: 5'-ggcgcttgcgtgcttccttg-3') and *B2M *(GenBank Accession No.: NM_004048, FP: 5'-agcgtactccaaagattcaggtt-3', RP: 5'-tacatgtctcgatcccacttaactat-3'). Specificity of PCR products obtained was characterized by melting-curve analysis followed by gel electrophoresis and DNA sequencing.

The following primers were used to amplify fragments for *in-situ *hybridization: *ATP1A3 *(GenBank Accession No.: NM_152296; FP: 5'-tcaagaaggaggtggctatg-3', RP: 5'-gagaagcagccagtgatgat-3');*NRGN *(GenBank Accession No.: NM_006176; FP: 5'-gactaggccagaactgagca-3', RP: 5'-agtggcacggagatgtagg-3'); *PVALB *(GenBank Accession No.: NM_002854; FP: 5'-agttgcaggatgtcgatgac-3', RP: 5'-ccagagtggagaattcgtca-3'); *ANXA2 *(GenBank Accession No.: NM_004039; FP: 5'-gatcatctgctccagaacca-3', RP: 5'-gagtcatacagccgatcagc-3').

### Comparison with SALS spinal-cord and AD hippocampus expression data

For SALS spinal-cord data, series GSE833 [4] of GEO was imported into Excel (Microsoft Corp.), and all UniGene ID numbers were updated (January 2006) using probe identities of the HuGeneFL array (Affymetrix, Inc.) in the NetAffx™ Analysis Center (Affymetrix, Inc.). Fold-changes were calculated for five SALS samples (GSM6827, GSM6828, GSM6834, GSM6835, GSM6836) vs. four control samples (GSM6826, GSM6829, GSM6830, GSM6831), for genes with a corresponding UniGene ID and showing a signal level of > 100 in at least five of these nine samples (3276 genes of a total of 7070 present on the HuGeneFL array). For 2266 of these genes the equivalent, quality-filtered (see above) non-redundant Agilent probes with the lowest uncorrected *P *value (see GenMAPP analysis above) were short-listed to calculate a correlation coefficient r for the expression ratios in spinal cord and motor cortex using Excel. A correlation coefficient was determined for all 2266 shared genes and for 23 shared genes corresponding to candidate genes identified in motor cortex by gene-based analysis, respectively.

For AD hippocampus data, an analogous procedure was followed, using seven severe-AD samples (GSM21203, GSM21206, GSM21207, GSM21208, GSM21212, GSM21213, GSM21229) vs. nine controls (GSM21215, GSM21217, GSM21218, GSM21219, GSM21220, GSM21221, GSM21226, GSM21231, GSM21232) of the GEO series GSE1297, and including genes showing a signal level of > 100 in at least eight of these 16 samples, resulting in a shared dataset of 6375 reliably detectable genes. A correlation coefficient was determined for the expression ratios diseased vs. control for these shared genes and for 38 shared genes identified as differentially expressed in our SALS study, respectively.

### Immunofluorescence

Tissue slices were submerged for 10 minutes in acetone at -20°C, followed by washes in phosphate-buffered saline (PBS), and incubation with 0.5% Triton in blocking solution (2% fetal calf serum, 2% bovine serum albumin, 0.2% fish-skin gelatine) for 10 minutes. Tissue slices were then incubated with 10-mM copper (II) sulfate and 50-mM ammonium acetate (pH 5.0) for 30 min to quench autofluorescence, followed by washes with PBS. Primary antibodies were co-incubated over-night at 4°C at dilutions of 1:50 for AQP1 (AQP1 antibody H-55, Santa Cruz Biotechnology, Inc.) and 1:3000 for neurofilaments (200 kDa + 160 kDa (phospho) antibody SMI 31, Abcam) in blocking solution without Triton X-100, followed by washes in PBS and co-incubation of secondary antibodies at room temperature at dilutions of 1:1500 anti-mouse Alexa Fluor 568 (A-11034, Molecular Probes) and 1:500 anti-rabbit Alexa Fluor 488 (A-21124, Molecular Probes). Tissue sections were then washed in PBS, incubated with Hoechst33342 as a DNA counter stain, washed in PBS, briefly air-dried, and mounted for microscopy in 10% Mowiol, 1% 1,4-Diazabicyclo [2.2.2]octane, and 25% glycerol in 0.1-M Tris buffer (pH 8). Images were acquired using an Axiovert 200 M microscope with the AxioVision 4.5 image acquisition software, the Apotome module and the transparency projection option for Z stacks of the Inside4D visualization module (all Carl Zeiss Inc.).

## Abbreviations

For Unigene cluster IDs of candidate genes see Figure 3, conforming to the HUGO Gene Nomenclature Committee (HGNC) database (see [135]). All abbreviations are defined at their first appearance in the text, and in the legends of tables and figures, as follows:

AD – Alzheimer's disease

ALS – amyotrophic lateral sclerosis

BP – biological process

CC – cellular component

EGL – external granular layer

EPL – external pyramidal layer

FALS – familial ALS

FP – forward primer

GO – gene ontology

IGL – internal granular layer

IPL – internal pyramidal layer

LMN – lower motoneuron

NF – neurofilament

MF – molecular function

MFL – multiform layer

ML – molecular layer

RP – reverse primer

qRT-PCR – quantitative reverse-transcriptase PCR

SALS – sporadic ALS

TCA – tricarboxylic acid cycle

UMN – upper motoneuron

## Authors' contributions

CWL participated in the design of the study, carried out microarray experiments, their pathway-based statistical analysis and comparisons with publicly available microarray data, participated in gene-based statistical analyses, performed qRT-PCR analyses, immunofluorescence experiments and statistical analysis of immunoblot data, and drafted the manuscript. AT performed in-situ hybridization experiments. MP performed immunoblots. NS conceived of the study, participated in its design and coordination, and helped to draft the manuscript. SC designed the study, participated in its coordination, performed gene-based statistical analysis of microarray data, statistical analysis of in-situ hybridization data, and made major contributions to the manuscript. All authors have read and approved the final manuscript.
